# High prevalence of low bone mineral density in wheelchair users regardless of sports participation: a cross-sectional analysis of the bonewheel study

**DOI:** 10.1007/s00421-025-06109-1

**Published:** 2025-12-24

**Authors:** Linn C. Risvang, Jan-Willem van Dijk, Julia K. Baumgart, Hannah M. Rice, Anja M. F. Liljegren, Vegard Strøm, Truls Raastad, Kristin L. Jonvik

**Affiliations:** 1 Department of Physical Performance, Norwegian School of Sport Sciences, Postboks 4014, Oslo, 0806 Norway; 2 Institute for Sports and Exercise Studies, HAN university of Applied Sciences, Nijmegen, The Netherlands; 3 SimArena, Department of Health and Functioning, Western Norway University of Applied Science, Bergen, Norway; 4 Department of Neuromedicine and Movement Science, Norwegian University of Technology and Science, Trondheim, Norway; 5 Department of Research, Sunnaas Rehabilitation Hospital, Nesoddtangen, Norway

**Keywords:** Spinal cord injury, Cerebral palsy, Spina bifida, Paraplegia, Bone health, Para sports, Lean body mass

## Abstract

**Purpose:**

Wheelchair users are at increased risk of low bone mineral density (BMD). While BMD varies by sport among Paralympic athletes, it remains unclear whether sports participation protects against low BMD in the impaired population. This study compared BMD at several skeletal sites between sports-active and non-sports-active wheelchair users.

**Methods:**

This study presents cross-sectional analyses of adult (18–60 years) wheelchair users with non-progressive impairments recruited for a subsequent multi-site intervention study. BMD and body composition were measured with dual-energy X-ray absorptiometry. Low BMD was defined as Z-score <–1.0. Multiple linear regression models assessed differences between sports-active (≥ 1 year organised; ≥3 training hours per week) and non-sports-active participants, and associations with impairment and exercise characteristics, and body composition.

**Results:**

Sixty-three wheelchair users (age 37 ± 11 years; 41% female; 48% sports-active; 49% with acquired impairments) were included. Low BMD was prevalent in 33%, 81% and 77% at the spine, hip and femoral neck, respectively. Low BMD at the hip was less prevalent in sports-active compared to non-sports-active (*p* = 0.013), without differences in any BMD Z-score models (all *p* > 0.05). Acquired impairments was positively associated with lumbar spine BMD (*p* = 0.034), while full-time wheelchair use was negatively associated with femoral neck BMD (*p* = 0.036). Impairments and SCIM scores were associated with hip BMD (both *p* < 0.05).

**Conclusion:**

Notably high prevalences of low BMD was found in this study, particularly at the hip and regardless of sports participation. Impairments and ambulatory ability appear the most influential on BMD. Targeted exercise interventions for bone health are warranted in this population.

**Trial registration:**

This study was registered in ClinicalTrials.gov (NCT05615402) on Nov 14, 2022, as well as Open Science Framework (10.17605/OSF.IO/SE2TB) on Jan 04, 2023.

**Supplementary Information:**

The online version contains supplementary material available at 10.1007/s00421-025-06109-1.

## Introduction

Wheelchairs users experience reduced mechanical loading on the skeleton compared to ambulatory individuals, who benefit from regular weight-bearing activities such as walking, running and jumping (Schäfer et al. 2023; Weston et al. 2017). Mechanical stimuli are essential for bone mass maintenance and primarily through dynamic loading and muscular forces that strain the bone tissue (Frost 2003; Mellon and Tanner 2012). Consequently, individuals who rely on wheelchairs for mobility are at increased risk of low bone mineral density (BMD), particularly in the lower body.

While physical activity and sports participation are generally considered beneficial for bone health, recent findings challenge this assumption in wheelchair users. Paralympic athletes, despite high training volumes, often present with clinically low BMD (Cavedon et al. 2021; Koivisto-Mørk et al. 2023; Weijer et al. 2024). These studies have found low-impact sports, such as hand biking and cross-country skiing, to be associated with low lumbar spine BMD. Even high-impact sports, like wheelchair basketball, rugby and tennis, show low BMD at the hip and femoral neck. Overall prevalences of low BMD were as high as 30–55% in wheelchair athletes in our recent study (Weijer et al. 2024). These findings suggests that sports participation alone may not mitigate the risk of low BMD in this population.

In the general population, weight-bearing physical activity is a cornerstone of osteoporosis prevention (Bae et al. 2023; Hong and Kim 2018; World Health Organization 2020). However, wheelchair users face unique barriers to engaging in such activities, limiting the mechanical stimuli necessary for bone remodelling in the spine, hips and lower limbs. Although adapted sports are available, their osteogenic potential remains poorly understood. Existing literature comparing BMD in wheelchair athletes and non-athletes are limited in scope, often focusing on males with a spinal cord injury (SCI) (Chain et al. 2012; Kopiczko and Cieplińska 2022; Teramoto 2010; Goktepe et al. 2004; Miyahara et al. 2008).

Moreover, the impact of different impairments on BMD is not fully elucidated. Individuals with SCI experience a rapid loss of bone post-injury due to neuromuscular inactivity, reduced mechanical loading, and altered neuroendocrine function (Antoniou et al. 2022; Choi et al. 2021; Haider et al. 2018; Jiang et al. 2006; Kelly et al. 2020). Other conditions such as cerebral palsy (CP), spina bifida, or lower-limb amputations may also put individuals at risk of low BMD, albeit through different mechanisms (Apkon et al. 2009; Kim et al. 2017). Importantly, the risk of osteoporosis may be compounded in populations living at higher latitudes, such as Norway, due to limited sun exposure and reduced endogenous vitamin D synthesis (Norwegian Institute of Public Health 2025).

At present, no study has comprehensively compared BMD at the lumbar spine, femoral neck and hip between sports-active and non-sports-active wheelchair users across a range of physical impairments and both sexes. Furthermore, the potential influence of sport type, particularly the impact level of the activity, on BMD remains unclear. Understanding these relationships is critical for developing targeted interventions to preserve bone health in this at-risk population.

Therefore, the primarily aim of this study was to compare BMD at both spine and hip between wheelchair users who participate in organised sports (sports-active) and those who do not (non-sports-active), in a cohort of Norwegian wheelchair users. We hypothesised that BMD would be lower in non-sports-active compared to sports-active wheelchair users, particularly at the hip. Secondary aims were to explore whether participation in high-impact activities is associated with higher BMD compared to low- or no-impact activities, and how participant characteristics, including impairment characteristics, impact on BMD.

## Methods and materials

### Study design and setting

A multi-centre cross-sectional design was utilised in this study, which also served as a screening process for recruiting eligible participants for a subsequent 24-week exercise and nutrition intervention (the ‘BoneWheel’ study (Risvang et al. 2025). Data were collected from December 2022 to November 2023. After a pre-screening telephone interview, the participants visited the research laboratories at the Norwegian School of Sport Sciences (Oslo, Norway), the Western Norway University of Applied Sciences (Bergen, Norway) or the Norwegian University of Technology and Science (Trondheim, Norway). The study was approved by the Regional Committee for Medical and Health Research Ethics of South-East Norway (REK 2023/458384) and was registered in ClinicalTrials.gov (NCT05615402) on Nov 14, 2022, as well as Open Science Framework (10.17605/OSF.IO/SE2TB) on Jan 04, 2023.

### Participants and recruitment

Sixty-seven wheelchair users were screened and categorised as sports-active or non-sports-active for the purpose of comparisons. Participants were classified as sports-active if they were currently participating in organised or competitive sports of any type and had maintained this activity for a minimum duration of one year and were currently training ≥ 3 h per week. The remaining participants were classified as non-sports-active. Further, all participants’ sport and exercise activities were classified as high-, low-, or no-impact based on biomechanical principles for loading of the lumbar spine and hip, through the typical movement pattern of different sports and activities and as previously described (Florvåg et al. 2025; Goktepe et al. 2004; Hilkens et al. 2023, 2025; Klomsten et al. 2018; Kopiczko et al. 2022; Miyahara et al. 2008; Turbanski and Schmidtbleicher 2010; Van Langendonck 2003; Weijer et al. 2024). This classification was completed by two authors (LCR, JKB) independently and any disagreement was solved through discussion. Agreement was 89% prior to discussion, whereby agreement reached 100%. Participant recruitment is described elsewhere (Risvang et al. 2025). In short, participants were recruited from both athletic and non-athletic environments. All invited participants received study information both orally and in writing and provided written informed consent prior to any study procedures in accordance with the Declaration of Helsinki.

#### Eligibility criteria

During recruitment and screening, participants were excluded from the present study only if (1) it was evident from a recruitment phone call interview that they did not match the eligibility criteria (Risvang et al. 2025) of the subsequent intervention study (consequently not invited to screening), or (2) if at any stage the participant did not match the present study eligibility criteria, which were: (i) 18–60 years of age, (ii) not post-menopausal, (iii) utilised a wheelchair for locomotion ≥ 50% of the time and (iv) any acquired impairments were minimum 2 years old (Antoniou et al. 2022). In general, for the present study and the intervention study, progressive neurological and muscular diseases and recent (within the last 5 years) medical treatment for low BMD excluded potential participants. Contrary to the subsequent intervention study, all levels of BMD Z-scores were included in the present study. Lastly, participants without a single valid DXA scans were excluded from the study.

### Study procedures

The participants attended the laboratory for a single visit, arriving after a minimum of 4-h fasting and 24 h of rest. Adherence to the preparations, including emptying the bladder, were registered and a dual energy X-ray absorptiometry (DXA) checklist probing contraindications (such as pregnancy) were completed, prior to DXA scans. Questionnaires were administered, collected, and processed digitally (SurveyXact, Xact by Rambøll, Denmark), and were completed by the participants prior to the visit. On a per need basis, paper questionnaires were filled instead.

### Measurements

#### Bone mineral density and body composition

Measurement procedures

Areal BMD (g/cm^2^) of the anterior-posterior (AP) lumbar spine, bilateral hips and femoral necks, as well as whole body composition were measured with the available DXA-system at each test site (Lunar iDXA or Prodigy, GE Healthcare, IL, USA; Horizon A, Hologic, MA, USA), as described elsewhere (Risvang et al. 2025). In short, the participants were scanned in a fasted state following morning urinary output. Daily calibration with the manufacturer-provided calibration block was performed. All procedures followed relevant manufacturer recommendations and or the International Society for Clinical Densitometry (ISCD) 2023 standard (The International Society for Clinical Densitometry 2023) and were performed by trained and extensively experienced operators (bioengineer/researchers/specialist nurse). Scans were checked for quality and re-taken during the same visit, when necessary (e.g., due to spasms) and possible, to improve scan quality. Scans with internal metal fixations or where large deformities made site measures impossible to analyse were excluded. All scan analyses were performed blinded by the main researcher (LCR) to avoid hypothesis driven bias. Scans from Lunar Prodigy and iDXA were analysed with enCORE enhanced analysis, while Hologic Horizon A scans were analysed in the Apex software (encore v. 18, GE Healthcare, IL, USA; Apex v.5.6.0.5, Hologic, MA, USA). All systems have previously shown high levels of precision in measuring both BMD (Ganda et al. 2014; Saarelainen et al. 2016; Watson et al. 2017) and body composition (Cheung et al. 2020; Henriksen et al. 2021; Vendrami et al. 2024). Precision assessments conducted at the main study site DXA-scanner (Lunar iDXA; enCORE version 18, GE Healthcare) confirmed similar precision in a similar group of wheelchair users (*n* = 12; three repeat scans within one week). All scans were performed and analysed by the same technologist (LCR). The ISCD Advanced precision analysis tool (ISCD 2025) was used to calculate the coefficients of variation (CV). CV of the BMD measurements were 1.4%, 4.1% and 4.4%, for lumbar spine, femoral neck and hip, respectively.

Anatomical sites measured

AP lumbar spine BMD was measured over first to fourth lumbar vertebra (L1–L4), however, mean values of L2–-L4 BMD is presented, as these were the vertebras with highest number of valid scans. Total hip and femoral neck were measured bilaterally, and the mean of both hips are reported when possible. In cases where only one side of the hip was valid (femoral neck *n* = 10 [18%], total hip *n* = 12 [21%]), that side is reported. Scans with artefacts (major spastic events, metal implants) that could not be omitted in the scan analysis were deemed invalid and excluded from total body BMD and BMC (*n* = 3; Hologic scans only). The scans were still included for body composition outcomes (lean body mass [LBM], FM and total mass). Where one upper extremity was not fully within the scan area, the specific limb segment was omitted, and the mirrored segment was duplicated automatically in the DXA software (‘estimated’; *n* = 13). However, in cases where both arms were registered as outside the scan area, and one arm was visually inspected to be negligibly lacking segment tissue area, this side was manually mirrored (*n* = 15). Consequently, whole-body values were recalculated (BMC, area, BMD, Z-scores, LBM, FM, and total mass).

DXA systems standardisation

To account for different DXA-systems used at the different test laboratories, all absolute BMD and bone mineral content (BMC), as well as LBM values obtained were standardised to Lunar iDXA values prior to further processing. The rational for standardisation was based on previous studies reporting significant differences between scanners (Ganda et al. 2014; Saarelainen et al. 2016; Vendrami et al. 2024; Watson et al. 2017), comparison of phantom and in-vivo measurements between the present study sites confirming similar differences in the same direction as previous reports (Prodigy vs. iDXA: +0–3%; Hologic vs. iDXA: – 10 to – 18%), as well as our group’s prior use of a similar strategy (Weijer et al. 2024). Values from scans taken on the Lunar Prodigy (*n* = 7) and Hologic Horizon A (*n* = 9) were standardised to Lunar iDXA with univariate correction equations (Ganda et al. 2014; Saarelainen et al. 2016; Vendrami et al. 2024; Watson et al. 2017). See Supplemental Materials Table S1 for details. BMD Z-scores were subsequently recalculated based on the iDXA-standardised BMD values and reference data from the National Health and Nutrition Examination Survey (NHANES) and the Lunar provided reference material (“Combined USA/NHANES” reference database, enCORE version 18 software; or manually with the USA/North Europe/NHANES references in the enCORE user manual (GE Healthcare 2020). As per the ISCD 2023 position, NHANES reference values were used for female femoral neck and both male and female total hip BMD Z-score calculations (The International Society for Clinical Densitometry 2023). Body mass correction was not utilised in this study due to unclear effect of body mass on BMD in this population (Weijer et al. 2024). BMD Z-scores were classified as osteopenia (Z-score < – 1.0) or osteoporosis (Z-score < – 2.0) according to the ISCD (The International Society for Clinical Densitometry 2023) and were used to assess the prevalence of these conditions in this study cohort.

#### Questionnaires

The participants responded to the Spinal Cord Independence Measure III for Self-Report (SCIM-SR) (Fekete et al. 2013) which evaluate the degree of everyday functional independence (i.e., transferring from the wheelchair to other surfaces and ambulation, as well as dressing and cleaning oneself). The overall score (out of 100) and the mobility subscale score (out of 41) are reported. Lastly, exercise and sports history, medical history and characteristics of the impairment and wheelchair use were also answered in a study specific background questionnaire.

### Data analysis and statistical reporting

Differences in BMD Z-scores across anatomical sites (lumbar spine, total hip, and femoral neck) were examined using repeated measures analysis of variance (ANOVA) with Greenhouse-Geisser correction for sphericity violations (ε = 0.609). Post hoc pairwise comparisons were adjusted with Bonferroni correction. Effect sizes are reported as partial η². Pearson correlation coefficients (r) were calculated to assess relationships between regional BMD Z-scores.

Rates of prevalent low BMD between sports-active and non-sports-active were compared with 2 × 2 contingency tables and Fisher’s exact test. To compare the continuous BMD Z-scores between sports-active and non-sports-active wheelchair users, linear regression analyses were performed. Separate models were constructed for each anatomical site. Multivariate models with backward selection included a priori selected independent variables with bivariate linear relationships with the dependent variable: impairment category (1: SCI; 2: CP; 3: spina bifida, 4: other) impairment onset (dichotomous; acquired or congenital), wheelchair use (dichotomous; full-time or part-time), SCIM scores (continuous; total score 0–100), age (continuous; years), height (continuous; cm), and LBM (continuous; kg), resistance exercise experience (continuous; years). Backward selection was applied to remove variables with negligible contribution to model fit based on non-significant R^2^ change (see Supplemental Table S2). Explores but excluded variables included years of wheelchair use, regular resistance exercise (≥ 2 sessions/week; yes/no), type and level of sport, other body composition variables (total body mass, FM, body mass index), number of medical prescriptions, and sex. Finally, sports-active (dichotomous; vs. non-sports-active) was added to the models, and adjusted R^2^ is provided for models with and without this variable (Table 3). Final models retained variables within 10% of the total number of observations.

Model assumptions (normality of residuals, homoscedasticity, multicollinearity, independence of errors) were evaluated and met (see Supplemental Table S3). Both unstandardized and standardized regression coefficients and standard error are presented. Significance level was set to α = 0.05. All statistical analyses were conducted using IBM Statistical Package for the Social Sciences (v. 28.0, IBM, NY, USA) or R studio (v2025.05.1 Build 513, Posit Software, PBC, MA, USA).

## Results

### Participants

Of 67 screened individuals, four were excluded, either due to simultaneously non-valid lumbar spine and hip scans (*n* = 2) or due to exclusion criteria (bisphosphonates treatment, *n* = 1; medical condition, *n* = 1). Thus, 63 wheelchair users were included in this study (37 ± 11 years; 41% female; 48% sports-active; 49% with acquired injuries, see Table 1 for all characteristics). The 30 sports-active wheelchair users had participated in their main sport for 16 ± 12 years, were currently training 7 ± 4 h per week and were competing at regional (*n* = 9), national (*n* = 10) and international level (*n* = 11; including one at the 2022 Paralympic Winter Games).

**Table 1:** Participant characteristics

Parameter	All *N* = 63	Non-sports-active *N* = 33	Sports-active *N* = 30
Age, years	37 ± 11	38 ± 10	35 ± 11
Sex, female/male, n (%)	26/37 (41/59)	16/17 (49/51)	10/20 (33/67)^*^
Height, cm^a^	167 ± 15	167 ± 14	166 ± 17
Total body mass, kg	69.6 ± 18.0	69.3 ± 17.6	70.0 ± 18.7
BMI, kg/m^2^	25 ± 5	25 ± 5	25 ± 6
Lean body mass, kg	40.9 ± 11.4	39.7 ± 10.3	42.2 ± 12.6
*Wheelchair dependency*			
Full-time WC use, n (%)	50 (79)	24 (73)	26 (87)
Years WC use	25 ± 13	26 ± 14	24 ± 13
*Impairments, n (%)*			
Spinal cord injury	26 (41)	15 (46)	11(37)
Years since injury	25 ± 9	26 ± 11	24 ± 7
Complete Paraplegia	12 (46)	6 (40)	6 (55)
Incomplete Paraplegia	8 (13)	4 (27)	4 (36)
Complete Tetraplegia	2 (8)	2 (13)	0
Incomplete Tetraplegia	4 (15)	3 (20)	1 (9)
Cerebral palsy	21 (33)	11 (33)	10 (33)
Spina bifida	7 (11)	3 (9)	4 (13)
Other^b^	9 (14)	4 (12)	5 (17)
*SCIM-SR*			
Mobility sub scale score (0–41)	9 ± 4	9 ± 4	10 ± 3
Total score (0–100)	70 ± 10	69 ± 9	71 ± 11
Resistance exercise 2x/week, n (%)	27 (43)	12 (38)	15 (56)
Weekly training hours	6 ± 5	2 ± 2	7 ± 4

Frequencies are given as % of column n, except spinal cord injury subcategories (% of spinal cord injuries)

*Abbreviations*: *BMI* body mass index, *WC* wheelchair, *SCIM-SR* spinal cord independence measure self-report

^a^Height was measured as stature (Seca 217 stadiometer, Seca GmbH & Co. KG., Germany) or as supine length depending on the participants ability to stand. Values are mean ± SD or frequencies (percentage)

^b^Other impairments: post-polio syndrome; lower limb amputation(s); arthrogryposis multiplex congenita; Ehlers-Danlos syndrome

*Indicate significant difference in sex distribution between sports-active and non-sports-active (*p* < 0.05)

Sports and exercise activities classified as high-impact for the spine were WC handball, rugby, basketball and tennis, sledge hockey, sit-volleyball, sit-ski, and resistance exercise (*n* = 28). Swimming, EL-bandy, sit-cross country skiing, arm cycling, curling, paddling and WC dancing were classified as low-impact sports and exercise activities (*n* = 12). Lastly, 23 were classified as no-impact activities or sedentary. For classification of sports impacting the hip, none were classified as high, however the same sports and exercise activities classified high for spine impact, were classified low for the hip (low-impact; WC handball, rugby, basketball and tennis, sledge hockey, sit-volleyball, sit-ski, and resistance exercise; *n* = 26), and those classified low- and no-impact (or sedentary) for the spine were combined for the hip (low- and no-impact; swimming, EL-bandy, sit-cross country skiing, arm cycling, curling, paddling and WC dancing, or sedentary; *n* = 37).

### Bone mineral density

**Fig. 1:**
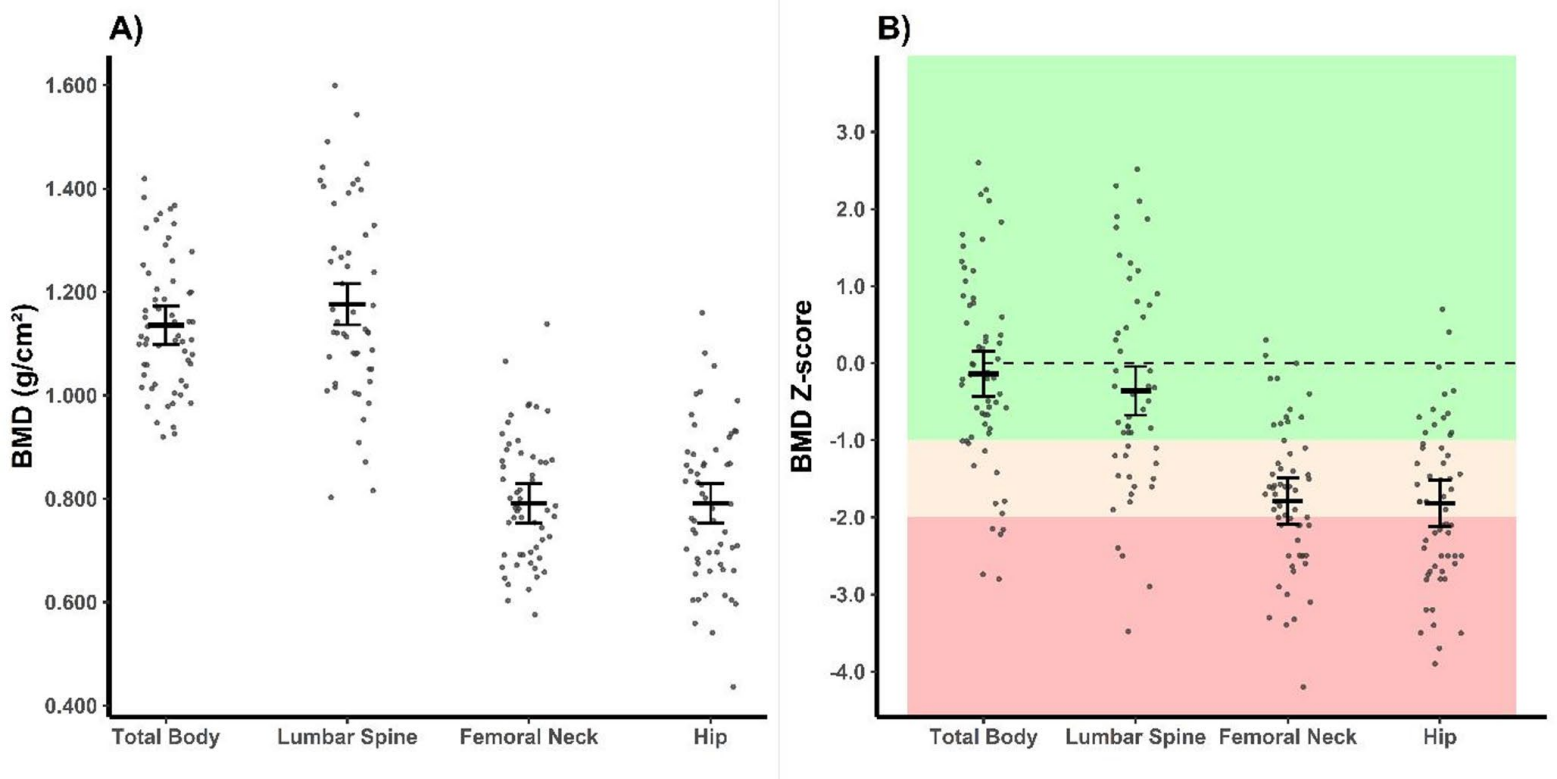
Whole-body and regional absolute BMD (**A**) and Z-scores (**B**) in Norwegian wheelchair users. Whole-body (*n* = 60), lumbar spine (*n* = 51), femoral neck (*n* = 57) and total hip BMD (*n* = 57) were measured with dual energy x-ray absorptiometry. Lumbar spine is measured over vertebra L2−L4, and unilateral or mean of bilateral femoral neck and hip is reported. Z-scores are standardised to sex and age-matched NHANES and/or Lunar USA/Northern Europe reference material (dashed line at Z-score = 0 represent reference population mean). Light and dark red indicate cut off Z-score values for low bone mass and osteoporosis, respectively, according to the World Health Organization and International Olympic Committee. Individual values shown as dots, horizontal line and error bars show estimated marginal means ± SE. *Abbreviations*: *BMD* bone mineral density. Created with R Studio

The overall mean BMD Z-scores were − 0.1 ± 1.3 SD at the whole-body (*n* = 60, 5% invalid scans), − 0.4 ± 1.4 SD at the L2-L4 lumbar spine (*n* = 51, 19% invalid scans), 1–.8 ± 1.0 SD at the total hip (*n* = 57, 10% invalid scans), and − 1.8 ± 0.9 SD at the femoral neck (*n* = 57, 10% invalid scans). The repeated measures ANOVA revealed significant differences between the anatomical sites (*F [1.2*,*51.1]* = 40.304, *p* < 0.001) with a partial η² = 0.490 indicating a large effect size. Pairwise comparisons revealed that BMD Z-score at the lumbar spine was significantly higher than at the femoral neck (mean difference [95% CI]: 1.3 [0.8, 1.9] SD, *p* < 0.001), and at the hip (1.3 [0.7–1.9] SD, *p* < 0.001; Fig. 1). No difference was seen between the femoral neck and hip (− 0.1 [− 0.4–0.3] SD, *p* = 1.000, Fig. 1). Low to no correlation between lumbar spine BMD Z-score and femoral neck or hip BMD Z-score was found (Pearson *r* = 0.31; *p* = 0.027, and *r* = 0.17; *p* > 0.05, respectively, see Supplemental Fig. S1). No sex differences were found in BMD Z-scores or in the prevalence of low bone mass (all *p* > 0.122). Overall, 93% of the participants had low BMD in at least one regional measurement site, while the prevalence at each anatomical site varied between 33 and 81%. Osteoporotic BMD Z-scores in at least one regional measurement site, was seen in 60% of the participants. Absolute BMD, Z-scores and prevalence of low BMD per region are presented in Table 2.

**Table 2:** Bone health amongst Norwegian sports-active and non-sports-active wheelchair users

	All *N* = 63	Non-sports-active *N* = 33	Sports-active *N* = 30	Fisher’s exact *p*-value
*Total body*				
BMD (g/cm^2^)	1.137 ± 0.127	1.130 ± 0.122	1.145 ± 0.135	> 0.05
BMD Z-score	– 0.1 ± 1.3	– 0.1 ± 1.2	– 0.1 ± 1.4	
Low BMD (%)	23	25	21	
Osteoporosis (%)	8	6	11	
Valid scans (%)	95	97	93	
*Lumbar spine (L2–L4)*				
BMD (g/cm^2^)	1.180 ± 0.189	1.147 ± 0.188	1.217 ± 0.187	> 0.05
BMD Z-score	– 0.3 ± 1.4	– 0.5 ± 1.4	– 0.1 ± 1.4	
Low BMD (%)	33	37	29	
Osteoporosis (%)	8	11	4	
Valid scans (%)	81	82	80	
*Femoral neck*				
BMD (g/cm^2^)	0.791 ± 0.130	0.766 ± 0.188	0.819 ± 0.155	> 0.05
BMD Z-score	– 1.8 ± 0.9	– 1.8 ± 0.9	– 1.7 ± 1.0	
Low BMD (%)	81	77	85	
Osteoporosis (%)	40	45	35	
Valid scans (%)	90	94	87	
*Total hip*				
BMD (g/cm^2^)	0.787 ± 0.148	0.749 ± 0.143	0.832 ± 0.144	**0.013**
BMD Z-score	– 1.8 ± 1.0	– 2.1 ± 0.9	– 1.6 ± 1.1	
Low BMD (%)	77	90	62	
Osteoporosis (%)	47	48	44	
Valid scans (%)	90	94	87	

Values are mean ± SD. Low BMD and osteoporosis refer to prevalence of BMD Z-score < – 1.0 and < – 2.0, as per ISCD Official Position 2023^5^. Lumbar spine is measured over vertebra L2–L4, and unilateral or mean of bilateral femoral neck and hip is reported

*Abbreviations*: *BMD* bone mineral density

#### Comparisons of BMD between sports-active and non-sports-active wheelchair users

**Fig. 2:**
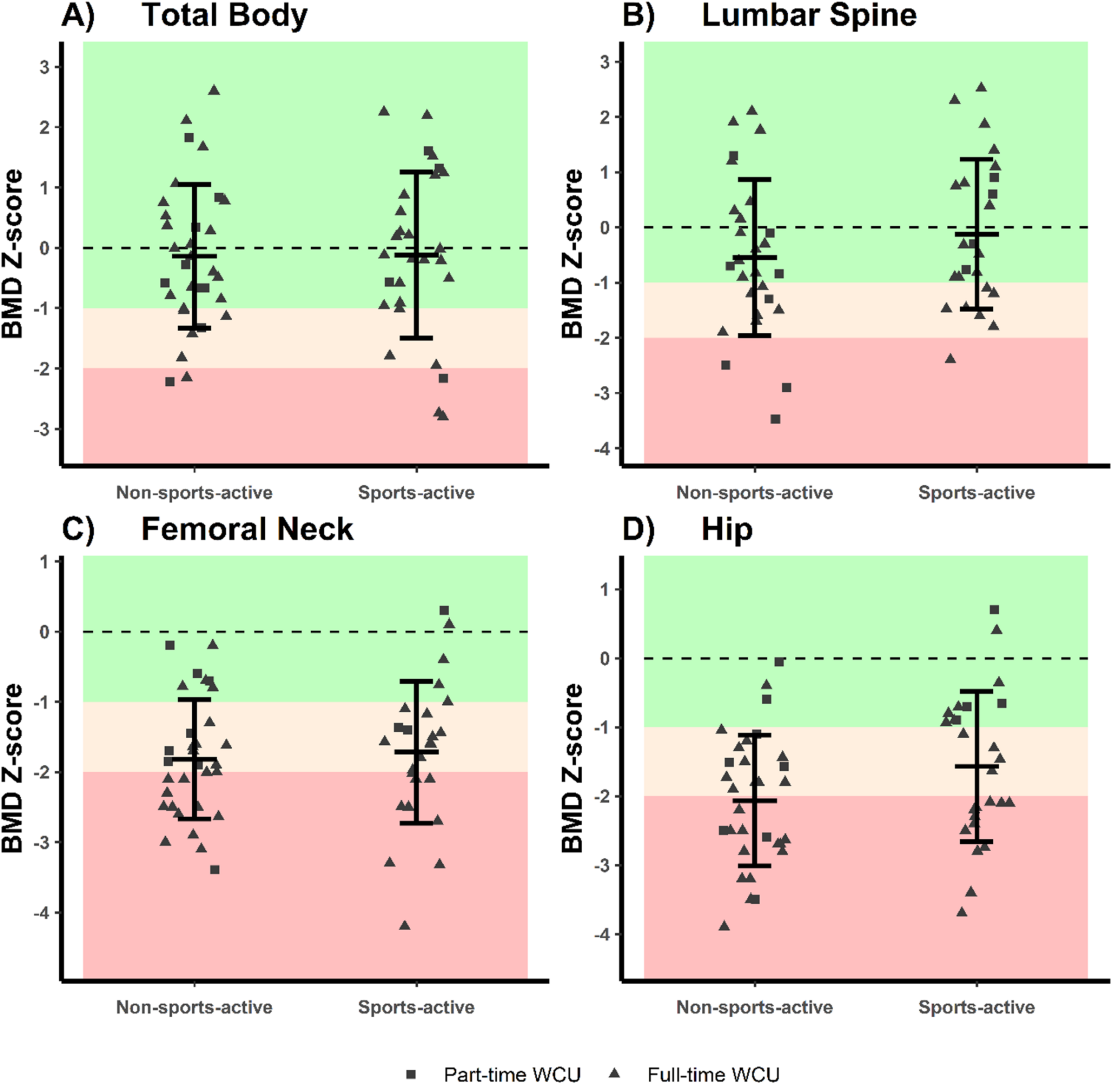
Total body (**A**) and regional (**B**–**D**) BMD Z-scores in sports-active (*n* = 30) and non-sports-active (*n* = 33) wheelchair users. Lumbar spine (**B**) is measured over vertebra L2-L4, and unilateral or mean of bilateral femoral neck (**C**) and hip (**D**) is reported. Z-scores are standardised to sex and age-matched NHANES and/or Lunar USA/Northern Europe reference material (dashed lines at Z-score = 0 SD represent the reference population mean). Light and dark red indicate cut off Z-score values for low bone mass and osteoporosis, respectively, according to the World Health Organization and International Olympic Committee. Error bars show mean ± SD. Abbreviations: BMD: bone mineral density. Created with R Studio

Prevalence of low BMD was significantly higher in non-sports-active than sports-active at the total hip (χ^2^ Fisher’s Exact test; *p* = 0.013; Table 2) but did not differ between sports-active and non-sports-active participants for lumbar spine, femoral neck or total body (χ^2^ Fisher’s Exact test: all *p* > 0.05; Table 2). There were no statistically significant differences in BMD Z-scores between sports-active and non-sports-active participants at any of the three anatomical sites after controlling for age and impairment onset for lumbar spine, age and full-time wheelchair use for femoral neck, or after controlling for impairment category and SCIM-SR score for hip BMD Z-score models (all *p* > 0.107, Fig. 2, Table 3A–C, Supplemental Table S2). Overall, the findings suggest physical function and ambulatory ability to be closest associated with BMD, irrespective of sports participation.

**Table 3:** Linear regression modelling of BMD Z-scores of (A) lumbar spine, (B) femoral neck and (C) total hip

(A)	Lumbar spine BMD Z-score	Unstandardized coefficients		Standardized coefficients				*R*^2^ change
		B	Std. Error	β	t	*p*- value	*R*^2^ adjusted	
Model						**0.003**	0.212	
(Constant)		-2.535	0.689		-3.677	**0.001**		
Impairment onset^a^		0.799	0.366	0.290	2.181	**0.034**		
Age, years		0.040	0.017	0.310	2.303	**0.026**		0.216^b^
Sports-active		0.585	0.352	0.212	1.662	0.103		0.044^c^

(B)	Femoral neck BMD Z-score	Unstandardized coefficients		Standardized coefficients	t			
		B	Std. Error	β		*p*-value	R^2^ adjusted	R^2^ change
Model						**0.033**	0.103	
(Constant)		-2.278	0.505		-4.506	**0.000**		
Age, years		0.025	0.011	0.280	2.177	**0.034**		
Full-time WC use^d^		-0.641	0.297	-0.278	-2.157	**0.036**		0.128^b^
Sports-active		0.284	0.239	0.155	1.186	0.241		0.023^c^

(C)	Total hip BMD Z-score	Unstandardized coefficients		Standardized coefficients	t			
		B	Std. Error	β		*p*-value	R^2^ adjusted	R^2^ change
Model						**0.006**	0.178	
(Constant)		-4.906	0.984		-4.987	**0.000**		
Impairment category^e^		0.249	0.123	0.256	2.027	**0.048**		
SCIM-SR score		0.034	0.014	0.313	2.484	**0.016**		0.183^b^
Sports-active		0.425	0.259	0.207	1.641	0.107		0.043^c^

Significance level was defined as *p*<0.05

*Abbreviations*: *BMD* bone mineral density, *SCIM-SR* spinal cord independence measure self-report, *WC* wheelchair

^a^Acquired vs. congenital impairment (reference)

^b^Model before inclusion of Sports-active; compared to unadjusted model

^c^Compared to^b^

^d^Compared to part-time use (i.e., some ambulatory ability)

^e^1: Spinal cord injury, 2: Cerebral palsy, 3: Spina bifida, 4: other (higher category number = higher BMD)

There were no significant differences in total body or lumbar spine BMD between high-, low- and no impact activities (both *p* > 0.05, Supplemental Fig. S2), nor were there any significant differences in femoral neck or hip BMD between high-impact and low-impact groups (both *p* > 0.05, Supplemental Fig. S3). BMD Z-scores across the represented sports is available in the Supplemental Information Fig. S4.

#### Other factors related to BMD

Table 3 shows unadjusted and adjusted effect estimates from the linear regression analyses, revealing that for the lumbar spine BMD Z-scores, acquired impairments and age were positively associated (both *p* < 0.05; Table 3A). For femoral neck BMD Z-scores, age was positively associated (*p* = 0.034), while full-time wheelchair use was negatively associated with BMD Z-scores (*p* = 0.036, Table 3B). At the total hip, impairment category (*p* = 0.048) and higher function (SCIM-SR score; *p* = 0.016) were positively associated with BMD Z-scores (Table 3C). For all variables in the backward selection, see Supplemental Table S3. Exploratory analysis of differences in BMD Z-scores between the four impairment categories were significant (multivariate ANOVA; regional BMD measure site x Impairment category; Wilk’s Lambda = 0.61, *F(2*,*38) = 12.044*, *p* < 0.001, partial η² = 0.263). Likely due to low statistical power, pairwise comparisons were not significant (all *p* > 0.05). However, upon visual inspection, those with spinal cord injury seemed to have higher lumbar spine estimated marginal mean BMD Z-scores than of those with CP (Supplemental Fig. S5). In line, the prevalences of low BMD at the lumbar spine were 10% and 59% in SCI and CP, respectively (Supplemental Table S4).

## Discussion

This study compared BMD at several skeletal sites between sports-active and non-sports-active wheelchair users and examined associations with participant characteristics. Overall, high rates of low BMD were observed, particularly at the hip and femoral neck. Lumbar spine was less affected and low correlation between the spine and hip BMD indicates the importance of multi-site assessment of BMD. While sports-active participants presented with lower prevalence of low BMD compared to non-sports-active, adjusted analyses showed no significant differences in BMD Z-scores between groups. Full-time wheelchair use and lower physical independence were associated with lower BMD at the femoral neck and hip, respectively, while type of impairment was important for both lumbar spine and total hip BMD.

### High prevalence of low bone mineral density

The prevalence of low BMD in this cohort was notably high, especially at the hip and femoral neck, where about 75–80% of participants exhibited Z-scores below − 1.0. This far exceeds rates reported in the general Norwegian population, where osteoporosis prevalence is estimated to around 5% (approximately 300,000 individuals in a population of 5.5 million; Norwegian Institute of Public Health 2025; Omsland et al. 2009). In contrast, about 60% of our participants met criteria for osteoporosis at one or more sites, underscoring the severity of skeletal fragility among wheelchair users. Our findings also differ from previous reports in impaired cohorts; for example, Smith et al. (2009) reported a 42% prevalence of hip osteopenia in adults with neurological impairments such as SCIs. However, the assessments were performed as early as three months post-injury. In the present study, all acquired impairments were at least 2 years old, a period after which bone loss typically begins to stabilize following the rapid decline in BMD seen in the subacute phase (Haider et al. 2018). These differences highlight the chronic nature of low BMD in long-term wheelchair users and the urgent need for tailored bone health strategies beyond those recommend for the general population (World Health Organization 2020).

In contrast to the pronounced deficits at the hip and femoral neck, lumbar spine BMD was less affected, with only 33% of participants classified with low BMD at this site. However, this finding should be interpreted with caution. Individuals with an SCI can potentially present with overestimated lumbar spine BMD due to factors such as vascular calcifications, degenerative changes and heterotopic ossifications (Biering-Sørensen et al. 1990; Leslie and Nance 1993; Bauman et al. 2009). Although we excluded about 20% of scans with visible artifacts, in line with recent findings (Ponzano et al. 2024), the potential for overestimation remains. The weak correlations between BMD between lumbar spine and hip (*r* = 0.31) and femoral neck BMD (*r* = 0.17) support the notion that bone loss in wheelchair users is site-specific and influenced by distinct mechanical and metabolic factors (Jiang et al. 2006). For instance, it seems that those who only partly use a wheelchair, i.e., also partly ambulate, have a stronger relationship between spine and femoral neck/hip BMD (Supplemental Fig. 1). Nonetheless, the findings highlight the importance of assessing multiple skeletal regions when evaluating bone health in this population.

### Sports participation and bone mineral density

Contrary to our hypothesis, we found no significant differences in BMD Z-scores between sports-active and non-sports-active participants. While the prevalence of low BMD at the hip was lower in the sports-active group (62% vs. 90%, *p* = 0.013), this did not translate into statistically significant differences in the adjusted linear regression model. These findings align with previous studies suggesting that adapted sports may not provide sufficient osteogenic stimulus, particularly at the hip and femoral neck (Koivisto-Mørk et al. 2023; Weijer et al. 2024). However, the present prevalence rates in the sports-active exceed those reported in previous studies of Paralympic athletes, suggesting that elite-level training may offer some protective effect. Although no statistically significant differences were observed between high-, low- and no-impact activity groups, visual inspection suggested that certain sports (i.e., wheelchair tennis, Supplemental Fig. S4) may be associated with higher lumbar spine BMD, which is in line with our previous study in Paralympic athletes (Weijer et al. 2024). However, this observation is based on a small subgroup and was not supported by statistical analysis and should therefore be interpreted with caution.

Importantly, the classification of participants into “sports-active” and “non-sports-active” groups may have limited our ability to detect meaningful differences. The sports-active group included individuals with a wide range of training volumes, sports, and performance levels, with only 12 participants competing at international level. Moreover, some non-sports-active individuals reported engaging in recreational physical activity such as resistance exercise, potentially narrowing the contrast between groups. Furthermore, classifying level of sports performance and osteogenic potential of the wheelchair sports and activities is a complicated task (McKay et al. 2021). Cautious interpretation due to the limitations of these classifications is recommended and further work to establish the mechanical loading of various adapted sports are warranted.

### Other factors associated with BMD

#### Physical function

Full-time wheelchair use was negatively associated with femoral neck BMD in this study, consistent with previous reports (Koivisto-Mørk et al. 2023; Smith et al. 2009; Weijer et al. 2024). At the hip, impairment category and lower SCIM-SR scores (indicating reduced physical independence) was associated with lower BMD Z-scores. These findings suggest that ambulatory ability may be a key determinant of hip BMD, likely due to the mechanical loading associated with standing and walking. Ambulation has previously indeed been reported as a significant positive predictor of hip BMD in both non-athletic (Smith et al. 2009) and athletic (Koivisto-Mørk et al. 2023; Weijer et al. 2024) impaired cohorts. Further, ~ 60% of those with acquired impairments in the present study, had an SCI, and it is known that those with chronic and particularly complete SCIs display little to no neuromuscular function below the injury level (World Health Organization 2020). This directly contributes to the disuse of the lower extremities. Thus, having a SCI or a spinal related impairment is an important risk factor of low BMD at the pelvis and lower extremities. However, it was not a significant factor compared to full-time wheelchair use when we explored spinal related impairments compared to non-spinal impairments as a potential predictor in this study. This may be due to lack of statistical power, or potentially other confounding factors such as body composition, as discussed above.

At the spine, acquired impairments was positively associated with higher lumbar spine BMD. This may seem contra intuitive; however, it likely reflects the impairments and its onset. Participants with acquired impairments (primarily SCI) sustained their injuries around age 25 years old, near peak bone mass (Weaver et al. 2016), whereas those with congenital impairments (primarily CP), typically start using a wheelchair at an early age. Thus, individuals with acquired injury have had the opportunity to accumulate higher bone mass through years of ambulation and non-impaired sports participation prior to impairment onset (Weaver et al. 2016). In contrast, those with congenital impairments may have used a wheelchair most of their lives and or present with neuro-muscular dysfunctions, including muscle spasms, diminished coordination, contractures and hypotonia, all of which limit the ability to perform weight-bearing activities. This restricted loading capacity contributes to low peak bone mass and increased skeletal fragility (Apkon et al. 2009; Kim et al. 2017).

#### Body composition

None of the measured body composition parameters were significantly associated with BMD in the final regression models. However, exploratory correlation analyses showed both total body mass and LBM to be modestly associated with lumbar spine BMD (Supplemental Table S2). Previous literature suggest that lower body mass and taller stature may be associated with increased fracture risk (Meyer et al. 1995; Lofthus et al. 2001). Furthermore, the association may be attenuated due to the range of impairments, representation of both sexes, regional body composition variations across impairments, and relatively high level of physical function in this cohort. LBM and muscle strength are closely linked (Janssen et al. 2000), and one may speculate that higher LBM may be indicative of a higher potential for bone remodelling stimuli through stronger muscle contractions. The findings are consistent with previous studies reporting modest associations between LBM and BMD in wheelchair users (Koivisto-Mørk et al. 2023; Weijer et al. 2024). Overall, the results of this study suggest that body composition has only a low to moderate association with BMD in wheelchair users.

#### Age

Age was positively associated with both lumbar spine and femoral neck BMD Z-scores in our cohort, which contrasts with typical patterns observed in non-impaired populations where BMD declines with age (Daly et al. 2013). This finding likely reflects methodological rather than biological factors. Z-scores are standardised against healthy reference populations (The International Society for Clinical Densitometry 2023), and older wheelchair users may appear relatively less impaired compared to age-matched controls who experience age-related bone loss. This finding underscores the need for caution when interpreting age effects and that Z-score BMD values may need additional age-adjustments in impaired cohorts.

### Strengths and limitations

This study included a diverse and representative sample of wheelchair users, with a range of impairments and representative sex distribution across impairments (60–70% males with SCI and CP; Raguindin et al. 2021; Himmelmann et al. 2009). The exclusion of individuals with recent injuries or progressive conditions reduced confounding from acute bone loss. We also collected detailed data on sports participation, physical function and body composition, allowing for a comprehensive analysis of BMD determinants.

However, several limitations should be acknowledged. The use of three different DXA-systems poses a methodological challenge, and although robust correction equations were applied, these have not been validated in populations with impairments or across both sexes (Saarelainen et al. 2016; Vendrami et al. 2024; Watson et al. 2017). Classifying participants as either sports-active or non-active is challenging, as there is no clear cut off in literature. Despite using several variables to classify each participant (sport participation, weekly training duration, competitiveness and training history), there is a range of performance levels within the sports-active and a range of impairments and total activity levels within both groups. The classification of sports by impact level was based on biomechanical principles of forces applied to the skeleton during various typical movements of the sports represented and were classified independently by two of the authors. Albeit others have also classified sports similarly (Florvåg et al. 2025; Goktepe et al. 2004; Hilkens et al. 2023, 2025; Miyahara et al. 2008; Turbanski and Schmidtbleicher 2010; Van Langendonck 2003; Weijer et al. 2024), the classification remains somewhat subjective.

Lastly, our statistical approach is a strength of this study. Using regression modelling is a robust statistical method, when statistical assumptions are met (e.g., normality of residuals and no dependency between independent variables) as well as total number of independent variables included in the models were well below 10% of observations. However, the modest sample size limited statistical power, and no a priori power calculations were performed due to this study being a screening process for a subsequent randomised controlled intervention study. Future studies may therefore want aim to recruit a larger cohort, as well as investigate other characteristics of sports participation’s relationship with BMD, such as quantifying degree of mechanical load from common movements in the adapted sports.

## Conclusions

Low BMD was highly prevalent among adult Norwegian wheelchair users, particularly at the hip and femoral neck, where over three-quarters of participants had Z-scores below − 1.0. Although sports-active individuals showed a lower prevalence of low BMD at the hip than non-sports-active participants, adjusted analyses revealed no significant differences in BMD Z-scores between groups. Instead, full-time wheelchair use, lower independence and impairments emerged as key determinants of regional BMD. These findings highlight the multifactorial nature of bone health in wheelchair users and the limited osteogenic effect of adapted sports alone. Future research should quantify mechanical loading in different sports and explore targeted strategies to preserve bone health. Large-scale, high-quality studies are needed to inform preventive and therapeutic interventions for this at-risk population.

## Supplementary Information

Below is the link to the electronic supplementary material.

Supplementary Material 1

## Data Availability

Deidentified data used in this study will be made available upon reasonable request.

## Abbreviations

ANOVA: Analysis of variance
BMC: Bone mineral content
BMD: Bone mineral density
CP: Cerebral palsy
CV: Coefficient of variation
DXA: Dual-energy X-ray absorptiometry
ISCD: International Society for Clinical Densitometry
LBM: Lean body mass
NHANES: National Health and Nutrition Examination Survey
SCIM-SR: Spinal cord independence measure III for self-report
SCI: Spinal cord injury
