# Supplementary material for: High prevalence of low bone mineral density in wheelchair users regardless of sports participation: a cross-sectional analysis of the bonewheel study
Source: Eur J Appl Physiol. 2025 Dec 24;126(5):2879–92. doi: 10.1007/s00421-025-06109-1 (PMC13236771; doi:10.1007/s00421-025-06109-1)
Supplement: Supplementary file 1 — Supplementary Material 1 [file 421_2025_6109_MOESM1_ESM.docx]

**Supplemental Materials**

for

**High prevalence of low bone mineral density in wheelchair users regardless of sports participation**: a cross-sectional analysis of the BoneWheel study

Linn C. Risvang^1^, Jan-Willem van Dijk^2^, Julia K. Baumgart^4^, Hannah M. Rice^1^, Anja M. F. Liljegren^3^, Vegard Strøm^1,5^, Truls Raastad^1^ and Kristin L. Jonvik^1^

*^1^Department of Physical Performance, Norwegian School of Sport Sciences, Oslo, Norway*

*^2^Institute for Sports and Exercise Studies, HAN university of Applied Sciences, Nijmegen, the Netherlands*

*^3^SimArena, Department of Health and Functioning,* *Western Norway University of Applied Science, Bergen, Norway*

*^4^Department of Neuromedicine and Movement Science, Norwegian University of Technology and Science, Trondheim, Norway*

*^5^Department of Research, Sunnaas Rehabilitation Hospital, Nesoddtangen, Norway*

**Address for correspondence**

| Dr. Kristin L. Jonvik |
| --- |
| Department of Physical Performance |
| Norwegian School of Sport Sciences |
| Postboks 4014 |
| 0806 Oslo, Norway |
| Tel: +47 23 26 23 69 |
| Email: kristinlj@nih.no |

**Table S1. Overview of correction equations used in standardising measurements between DXA-systems.**

| Variable | Standardised to iDXA | Lunar | | Hologic | |
| --- | --- | --- | --- | --- | --- |
|  |  | Prodigy equation ^a^ | p-value ^b^ | Horizon A equation | p-value ^b^ |
| BMD, g/ cm^2^ |  |  |  |  |  |
| L2-L4 spine | P + H | 0.008+0.986*BMD_P_  (Watson et al. 2017) | >0.05 | −0.06+1.24*BMD_H_  (Ganda et al. 2014) | <0.001 |
| Femoral neck | P + H | 1.0050*BMD_P_  (Saarelainen et al. 2016) | <0.05 | 0.06+1.12*BMD_H_  (Ganda et al. 2014) | 0.012 |
| Total hip | P + H | 1.0095*BMD_P_  (Saarelainen et al. 2016) | <0.05 | −0.02+1.08*BMD_H_  (Ganda et al. 2014) | 0.026 |
| Whole-body | P | 0.035+0.965*BMD_P_  (Watson et al. 2017) | <0.05 | *Not published* | na |
| Whole-body composition | |  |  |  |  |
| BMC, g | H | 0.017+1.003*BMC_P_  (Watson et al. 2017) | <0.05 | 80.4+1.104*BMC_H_  (Vendrami et al. 2024) | <0.001 |
| Area, cm^2^ | P | 112+0.966*BA_P_  (Watson et al. 2017) | <0.05 | *Not published*  (Vendrami et al. 2024) | <0.001 |
| LBM, g | H | -0.262+1.029*LBM_P_  (Watson et al. 2017) | >0.05 | 5477.8+0.826∗LBM_H_  (Vendrami et al. 2024) | <0.001 |

^a^ Prodigy enhanced analysis was performed prior to standardisation.

^b^ P-values of statistical testing of absolute differences between GE Lunar iDXA and DXA-system in the cited publications.

**Abbreviations:** P: GE Lunar Prodigy, H: Hologic Horizon, na: not available.

**Table S2. Backward selection of variables to final linear regression BMD Z-score models.**

| **Lumbar spine** | **Variables Entered** | **Variables Removed** | **R** | **R Square** | **Adjusted R Square** | **Std. Error of the Estimate** | **R Square Change** | **F Change** | **df1** | **df2** | **Sig. F Change** |
| --- | --- | --- | --- | --- | --- | --- | --- | --- | --- | --- | --- |
| 1 | Acquired, RE (years), Full-time WC use, Height (cm), Age (years), Total LBM (kg) |  | 0.58 | 0.34 | 0.24 | 1.3 | 0.34 | 3.444 | 6 | 40 | **0.008** |
| 2 |  | Total LBM (kg) | 0.58 | 0.34 | 0.25 | 1.2 | 0.00 | 0.287 | 1 | 40 | 0.595 |
| 3 |  | RE (years) | 0.56 | 0.32 | 0.25 | 1.2 | -0.02 | 1.123 | 1 | 41 | 0.296 |
| 4 |  | Acquired | 0.55 | 0.30 | 0.25 | 1.3 | -0.02 | 1.303 | 1 | 42 | 0.260 |
| **Femoral neck** | **Variables Entered** | **Variables Removed** | **R** | **R Square** | **Adjusted R Square** | **Std. Error of the Estimate** | **R Square Change** | **F Change** | **df1** | **df2** | **Sig. F Change** |
| 1 | SCIM-SR mobility score, Age (years), Full-time WC |  | 0.40 | 0.16 | 0.10 | 0.87 | 0.16 | 3.02 | 3 | 49 | **0.038** |
| 2 |  | SCIM-SR mobility score | 0.39 | 0.15 | 0.12 | 0.86 | 0.00 | 0.15 | 1 | 49 | 0.703 |
| **Total hip** | **Variables Entered** | **Variables Removed** | **R** | **R Square** | **Adjusted R Square** | **Std. Error of the Estimate** | **R Square Change** | **F Change** | **df1** | **df2** | **Sig. F Change** |
| 1 | RE (years), SCIM score, Impairment category, Full-time WC use |  | 0.47 | 0.221 | 0.154 | 0.89 | 0.221 | 3.267 | 4 | 46 | **0.019** |
| 2 |  | RE (years) | 0.45 | 0.205 | 0.154 | 0.89 | -0.016 | 0.971 | 1 | 46 | 0.33 |
| 3 |  | Full-time WC use | 0.42 | 0.177 | 0.143 | 0.90 | -0.028 | 1.654 | 1 | 47 | 0.205 |

Backward selection criterion: Probability of F-to-remove ≥0.100. Abbreviations: RE: resistance exercise, WC: wheelchair, LBM: lean body mass, SCIM-SR: spinal cord independence measure – self-report, BMI: body mass index.

**Table S3. Regression Diagnostics for Assumption Checks.**

| **Assumption** | **Lumbar Spine** | **Total Hip** | **Femoral Neck** |
| --- | --- | --- | --- |
| Linearity | Residuals appear randomly scattered | Residuals appear randomly scattered | Residuals appear randomly scattered |
| Homoscedasticity | Std. Residuals: ±2.07; SD ≈ 0.97 | Std. Residuals: ±2.77; SD ≈ 0.97 | Std. Residuals: ±2.51; SD ≈ 0.96 |
| Normality of Residuals | Residual Mean ≈ 0; Symmetric distribution | Residual Mean ≈ 0; Symmetric distribution | Residual Mean ≈ 0; Symmetric distribution |
| Multicollinearity | Tolerance > 0.94 for all predictors | Tolerance > 0.93 for all predictors | Tolerance > 0.94 for all predictors |
| Influential Points | Std. Predicted ±2.34 | Std. Predicted ±2.56 | Std. Predicted ±2.47 |
| Independence of Errors | Durbin-Watson = 2.35; no autocorrelation detected | Durbin-Watson = 1.90; no autocorrelation detected | Durbin-Watson = 1.8–2.5; no autocorrelation detected |

Linearity: Assessed visually via residual plots. Random scatter around zero suggests the linearity assumption is met.

Homoscedasticity: Standardized residuals within ±3 and relatively consistent spread across predicted values suggest no major heteroscedasticity.

Normality of Residuals: Mean residuals close to zero and symmetric distribution (based on residual statistics) support normality.

Multicollinearity: Tolerance values > 0.90 indicate low multicollinearity among predictors.

Influential Points: Standardized predicted values within ±3 suggest no extreme outliers or leverage points.

Independence of Errors: Durbin-Watson test was not included in the SPSS output; assumption not formally tested.

**Table S4. Absolute BMD and BMD Z-scores by diagnosis.**

|  |  | | L2-L4 BMD | Femoral neck BMD | Total hip BMD | Total Body BMD | L2-L4 Z-score | Femoral neck Z-score | Total Hip Z-score | Total Body Z-score |
| --- | --- | --- | --- | --- | --- | --- | --- | --- | --- | --- |
| All | N | Valid | 51 | 58 | 57 | 60 | 51 | 57 | 57 | 60 |
|  |  | Missing | 12 | 5 | 6 | 3 | 12 | 6 | 6 | 3 |
|  | Mean | | 1.180 | 0.791 | 0.787 | 1.137 | -0.35 | -1.77 | -1.84 | -0.13 |
|  | Std. Deviation | | 0.189 | 0.130 | 0.148 | 0.127 | 1.39 | 0.92 | 1.04 | 1.27 |
| SCI | N | Valid | 21 | 26 | 25 | 24 | 21 | 26 | 25 | 24 |
|  |  | Missing | 5 | 0 | 1 | 2 | 5 | 0 | 1 | 2 |
|  | Mean | | 1.261 | 0.796 | 0.758 | 1.170 | 0.25 | -1.80 | -2.11 | 0.00 |
|  | Std. Deviation | | 0.181 | 0.116 | 0.124 | 0.131 | 1.30 | 0.84 | 0.81 | 1.12 |
| CP | N | Valid | 17 | 18 | 17 | 20 | 17 | 17 | 18 | 20 |
|  |  | Missing | 4 | 3 | 4 | 1 | 4 | 4 | 3 | 1 |
|  | Mean | | 1.107 | 0.788 | 0.790 | 1.090 | -0.90 | -1.48 | -1.70 | -0.44 |
|  | Std. Deviation | | 0.150 | 0.143 | 0.151 | 0.108 | 1.04 | 0.88 | 1.27 | 1.37 |
| SB | N | Valid | 5 | 6 | 7 | 7 | 5 | 6 | 6 | 7 |
|  |  | Missing | 2 | 1 | 0 | 0 | 2 | 1 | 1 | 0 |
|  | Mean | | 1.109 | 0.786 | 0.833 | 1.185 | -1.03 | -2.61 | -1.84 | 0.36 |
|  | Std. Deviation | | 0.304 | 0.222 | 0.246 | 0.164 | 2.07 | 1.39 | 1.29 | 1.72 |
| Other | N | Valid | 8 | 8 | 8 | 9 | 8 | 8 | 8 | 9 |
|  |  | Missing | 1 | 1 | 1 | 0 | 1 | 1 | 1 | 0 |
|  | Mean | | 1.168 | 0.783 | 0.830 | 1.116 | -0.31 | -1.66 | -1.30 | -0.18 |
|  | Std. Deviation | | 0.141 | 0.077 | 0.112 | 0.106 | 1.39 | 0.51 | 0.77 | 1.05 |

Abbreviations: BMD: bone mineral density, SCI: Spinal cord injury, CP: cerebral palsy, SB: spina bifida.

**
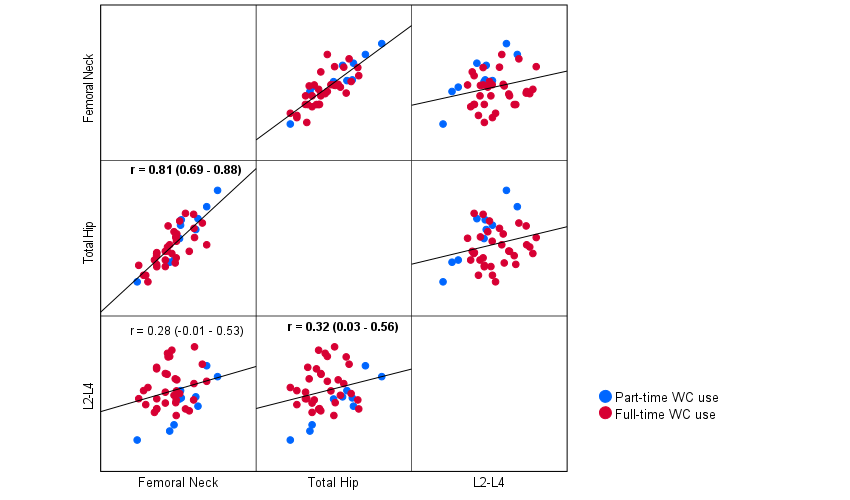
**

**Figure S1. Relationships between regional BMD Z-scores by wheelchair use.** Lumbar spine was measured over vertebra L2-L4. Unilateral or mean of bilateral femoral neck and hip is reported. Z-scores are standardised to sex and age-matched NHANES and/or Lunar USA/Northern Europe reference material. Pearson r correlation coefficients for the whole cohort and 95% CI are presented. Created with SPSS.


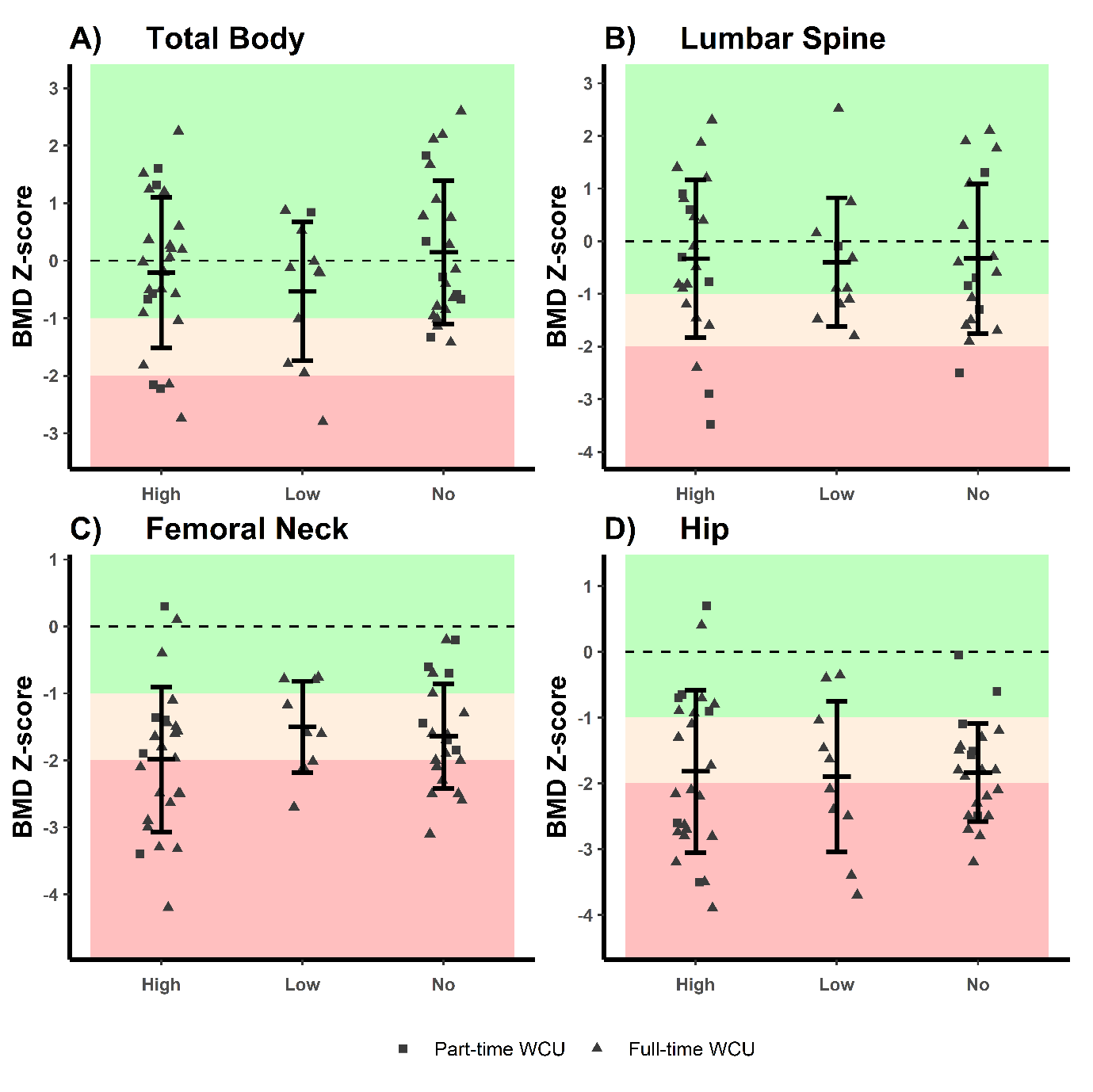


**Figure S2. Total body and lumbar spine BMD Z-score across sports and activity impact.** Lumbar spine was measured over vertebra L2-L4. High-impact sports: resistance exercise, WC handball, basketball, rugby and tennis, sit-volleyball, sledge hockey, and alpine sit-ski (n=28). Low-impact sports: WC dancing, sit-cross country skiing, EL-bandy, arm cycling, curling, paddling and swimming (n=12). No impact activities or sedentary: n=23. Z-scores are standardised to sex and age-matched NHANES and/or Lunar USA/Northern Europe reference material. Light and dark red indicate cut off Z-score values for low bone mass and osteoporosis, respectively, according to the World Health Organisation and International Olympic Committee. Individual values shown as triangles (full-time WC user) and squares (part-time WC user), while error bars show mean ± SD. Abbreviations: WC: wheelchair. Created with R Studio.


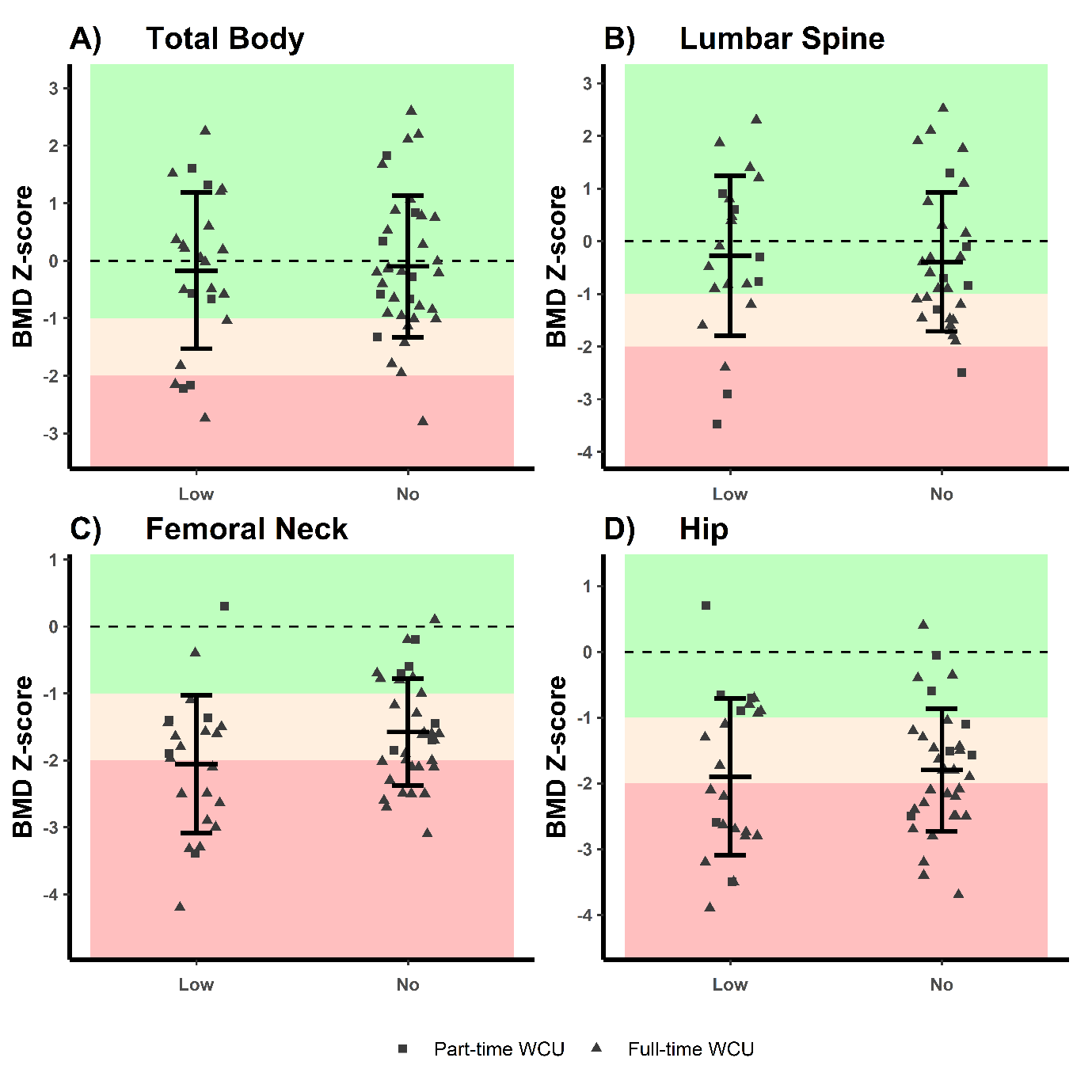


**Figure S3. Hip and femoral neck BMD Z-score across sports and activity impact.**

Low-impact sports: resistance exercise, WC handball, basketball, rugby and tennis, sit-volleyball, sledge hockey, and alpine sit-ski were classified as and activity (n=26). No impact activity or sedentary: n=37. Z-scores are standardised to sex and age-matched NHANES and/or Lunar USA/Northern Europe reference material. Light and dark red indicate cut off Z-score values for low bone mass and osteoporosis, respectively, according to the World Health Organisation and International Olympic Committee. Individual values shown as triangles (full-time WC user) and squares (part-time WC user), while error bars show mean ± SD. Created with R Studio.

**
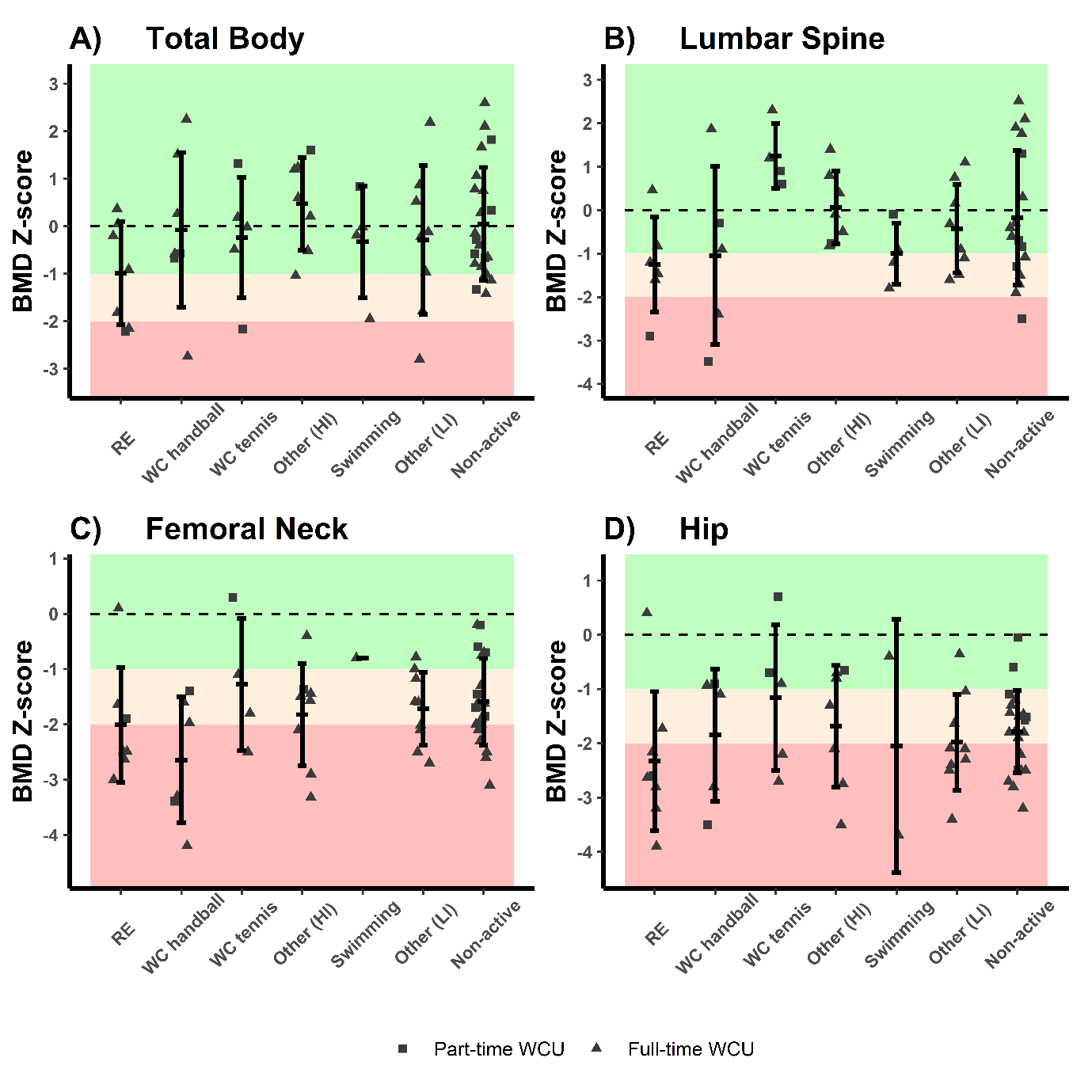
**

**Figure S4. Whole-body and regional BMD Z-score across represented sports.** Lumbar spine is measured over vertebra L2-L4, and unilateral or mean of bilateral femoral neck and hip is reported. Other high-impact (HI) sports: WC rugby and basketball, sledge hockey, sit-volleyball, alpine sit-ski, functional fitness. Other low-impact (LI) sports: arm cycling, WC dancing, para-curling, seated cross-country skiing, EL-bandy, boccia, and shooting. Z-scores are standardised to sex and age-matched NHANES and/or Lunar USA/Northern Europe reference material. Light and dark red indicate cut off Z-score values for low bone mass and osteoporosis, respectively, according to the World Health Organisation and International Olympic Committee. Individual values shown as triangles (full-time WC user) and squares (part-time WC user), while error bars show mean ± SD. *Created with R Studio.*


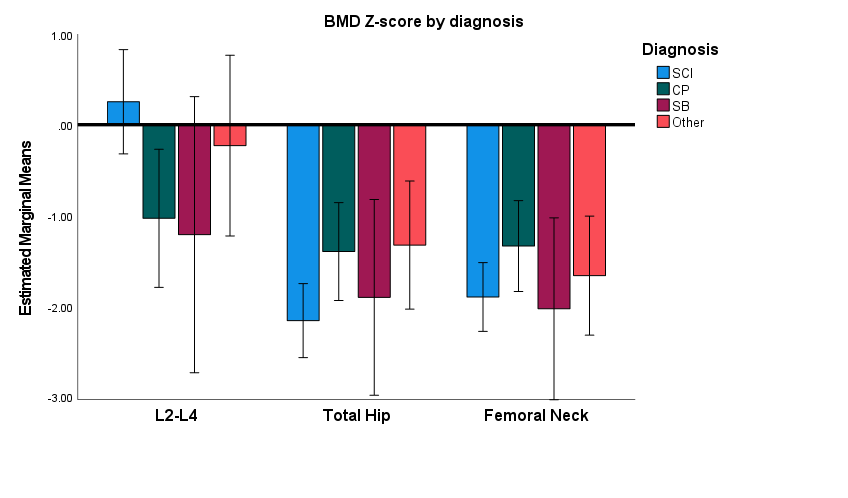


**Figure S5. Estimated marginal mean BMD Z-scores by diagnosis.** Error bars represent 95% confidence interval. SCI: spinal cord injury, CP: cerebral palsy, SB: spina bifida. *Created with SPSS.*
